# Supplementary material for: Ligand efficacy modulates conformational dynamics of the µ-opioid receptor
Source: Nature. 2024 Apr 10;629(8011):474–80. doi: 10.1038/s41586-024-07295-2 (PMC11078757; doi:10.1038/s41586-024-07295-2)
Supplement: Supplementary file 1 — Supplementary Figs. 1–8. [file 41586_2024_7295_MOESM1_ESM.pdf]

nature portfolio

https://doi.org/10.1038/s41586-024-07295-2

Supplementary information

Ligand efficacy modulates conformational dynamics of the µ-opioid receptor

In the format provided by the authors and unedited

Supplementary information for

Ligand efficacy modulates conformational dynamics of the µ-opioid receptor

Authors: Jiawei Zhao[1,2]†, Matthias Elgeti[3,4]†*, Evan S. O'Brien[5], Cecília P. Sár[6], Amal EI Daibani[7], Jie Heng[1,2], Xiaoou Sun[1,2], Elizabeth White[5], Tao Che[7], Wayne L. Hubbell[3], Brian K. Kobilka[5]*, Chunlai Chen[1,8]*

Affiliations:

[1]State Key Laboratory of Membrane Biology, Beijing Advanced Innovation Center for Structural Biology, Tsinghua University; Beijing, 100084, China.

[2]Tsinghua-Peking Joint Center for Life Sciences, School of Medicine, Tsinghua University; Beijing, 100084, China.

[3]Jules Stein Eye Institute and Department of Chemistry and Biochemistry, University of California; Los Angeles, Los Angeles, CA 90095, USA.

[4]Institute for Drug Discovery, University of Leipzig Medical Center; 04107 Leipzig, Germany.

[5]Department of Molecular and Cellular Physiology, Stanford University School of Medicine; Stanford, CA 94305, USA.

[6]Institute of Organic and Medicinal Chemistry, School of Pharmaceutical Sciences, University of Pécs; Szigeti st. 12, H-7624 Pécs, Hungary.

[7]Department of Anesthesiology, Washington University School of Medicine; Saint Louis, MO 63110, USA

[8]School of Life Sciences, Tsinghua University; Beijing, 100084, China.

†These authors contributed equally to this work.

*Corresponding author. Email: matthias.elgeti@uni-leipzig.de (M.E.); kobilka@stanford.edu (B.K.K.); chunlai@mail.tsinghua.edu.cn (C.C.)

Table of contents

Supplementary Fig. 1. Ligands used in this study. 3

Supplementary Fig. 2. Synthesis of HO-1427. 4

Supplementary Fig. 3. DEER dipolar evolution data and 6-Gaussian model-based fits. 5

Supplementary Fig. 4. Representative fluorescence traces of Cy3 and Cy5 labeled µORΔ7-182C/273C (µOR-Cy3/Cy5)... 6

Supplementary Fig. 5. Exemplary smFRET traces and transitions of µOR-Cy3/Cy5 in the presence of 20 µM with different GDP concentrations. 7

Supplementary Fig. 6. Fitting high-FRET dwell time. 8

Supplementary Fig. 7. Fitting low-FRET dwell time.. 9

Supplementary Fig. 8. The raw, uncropped gel images for Extended Data Fig. 3a 10

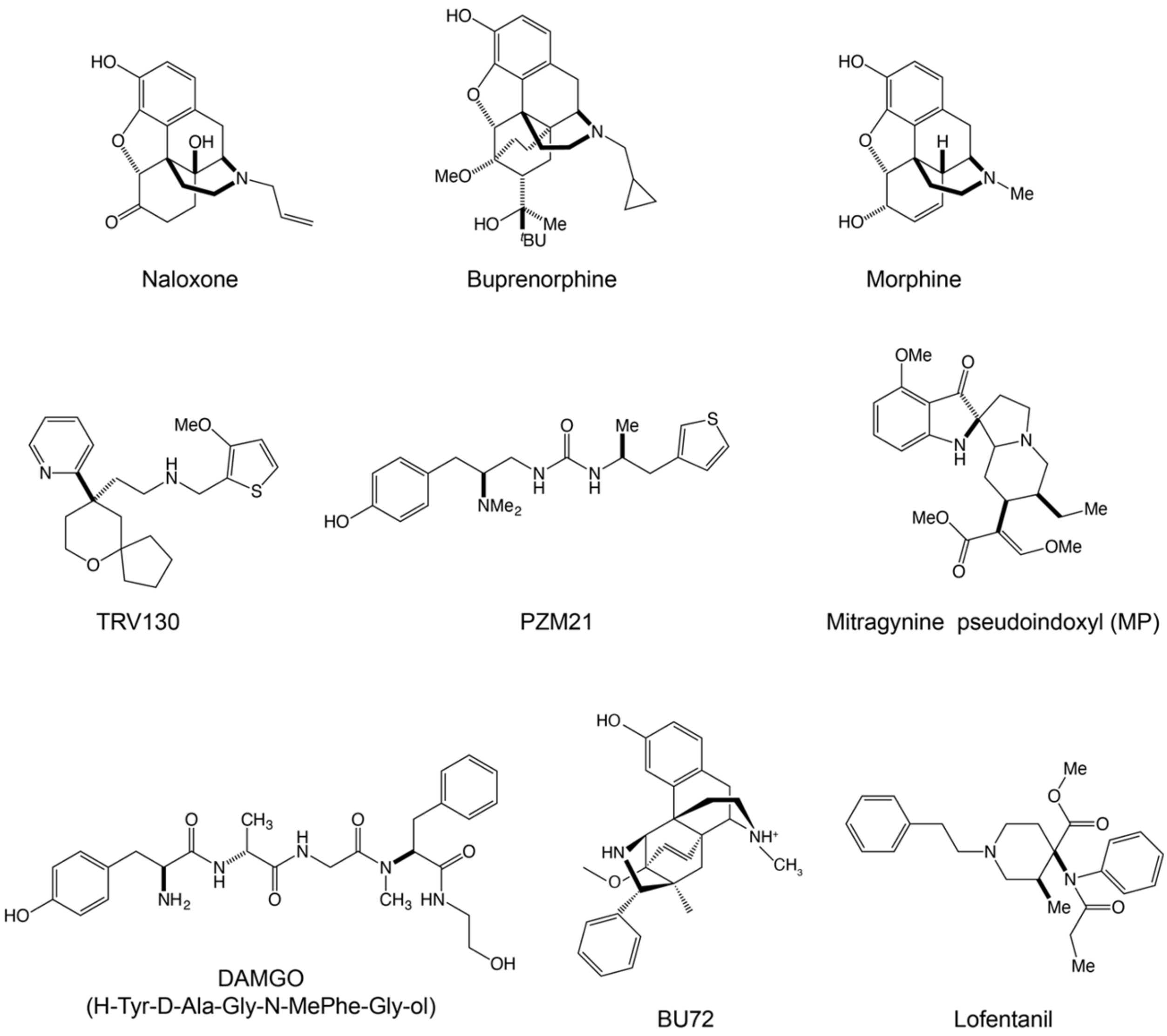

Supplementary Fig. 1. Ligands used in this study.

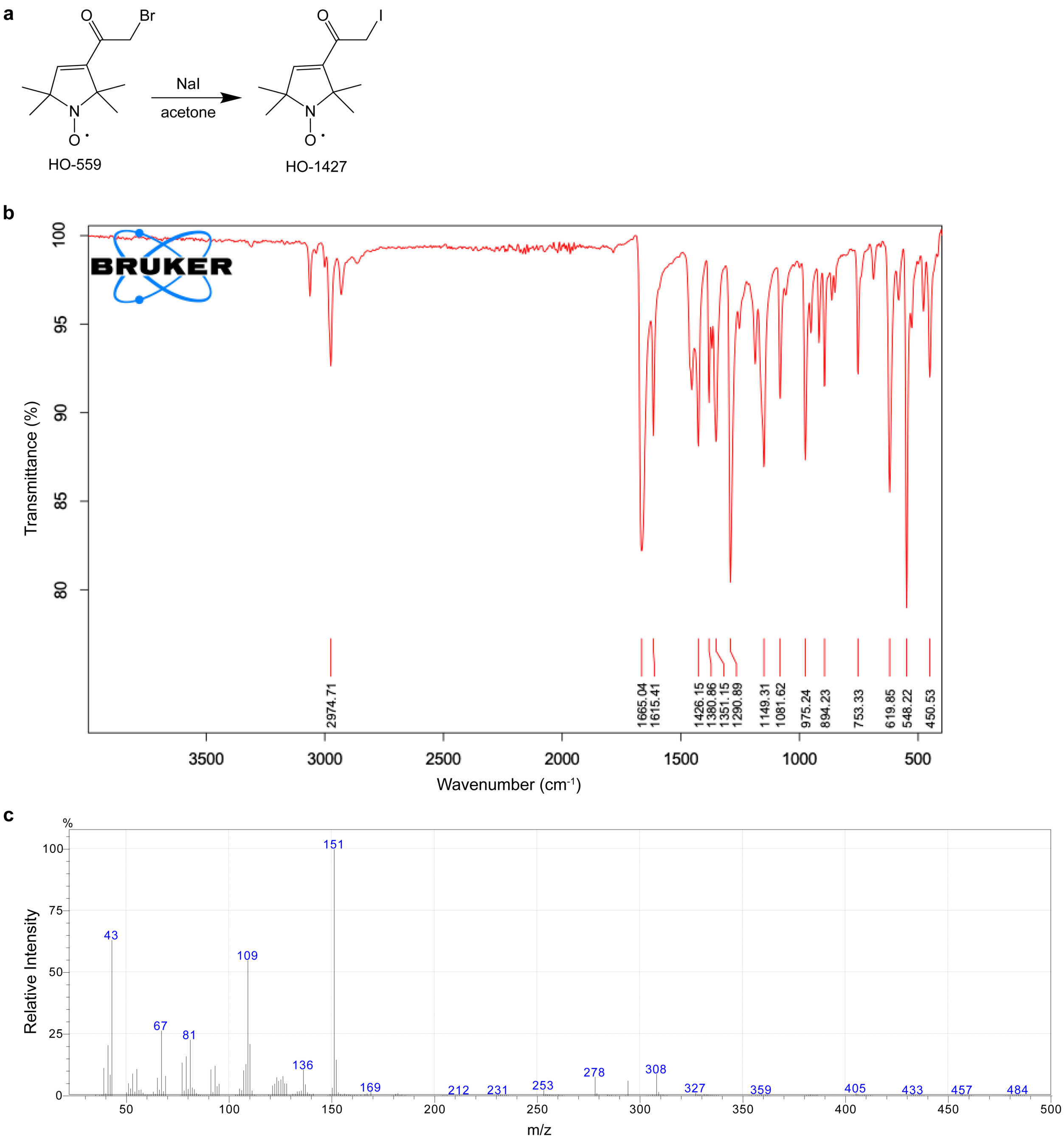

Supplementary Fig. 2. Synthesis of HO-1427. a, Schematic of HO-1427 synthesis. **b**, Fourier-transform infrared spectroscopy (FTIR) of HO-1427. **c**, Mass spectrum of HO-1427.

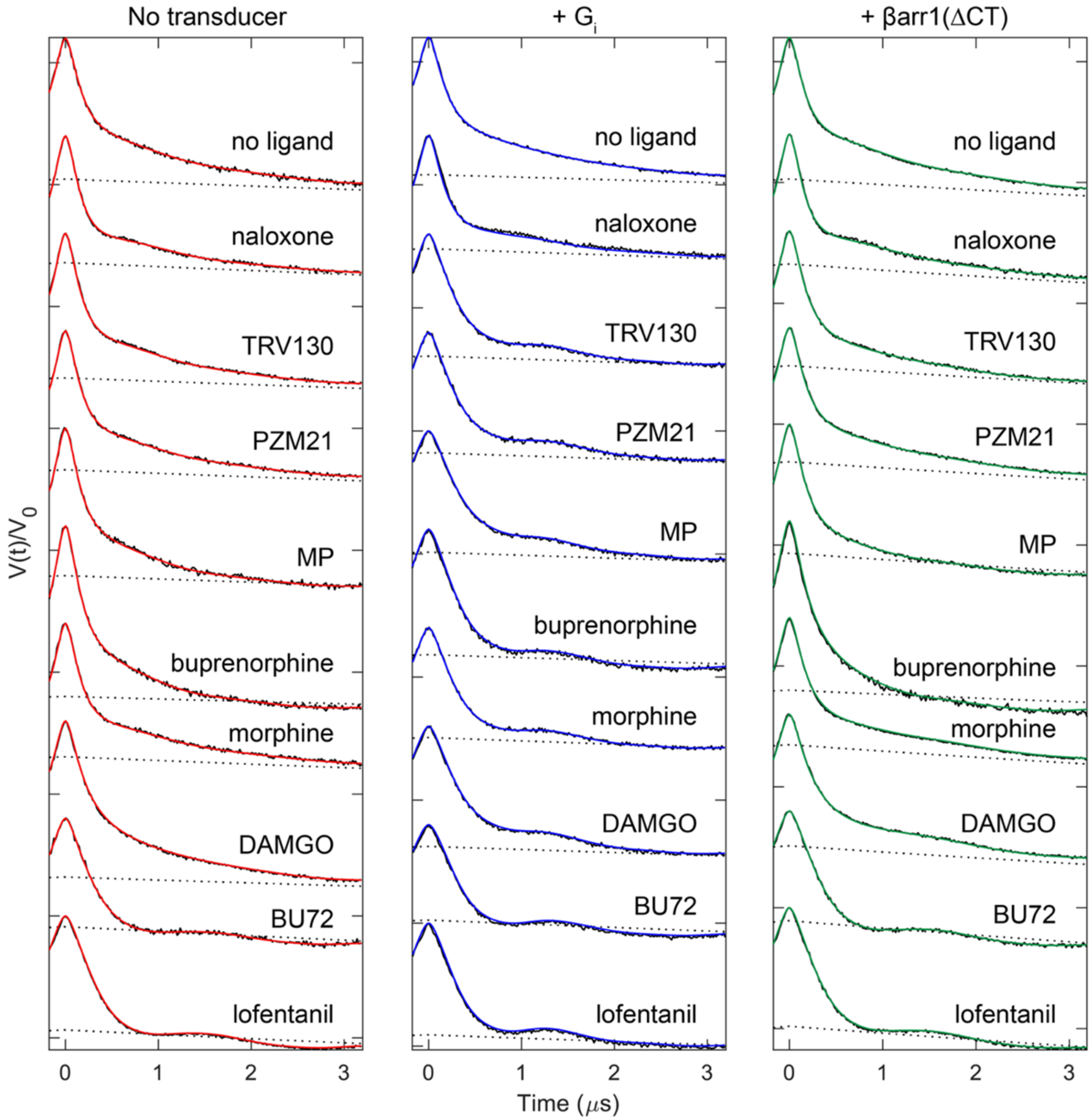

Supplementary Fig. 3. DEER dipolar evolution data and 6-Gaussian model-based fits. Dotted lines indicate background signal.

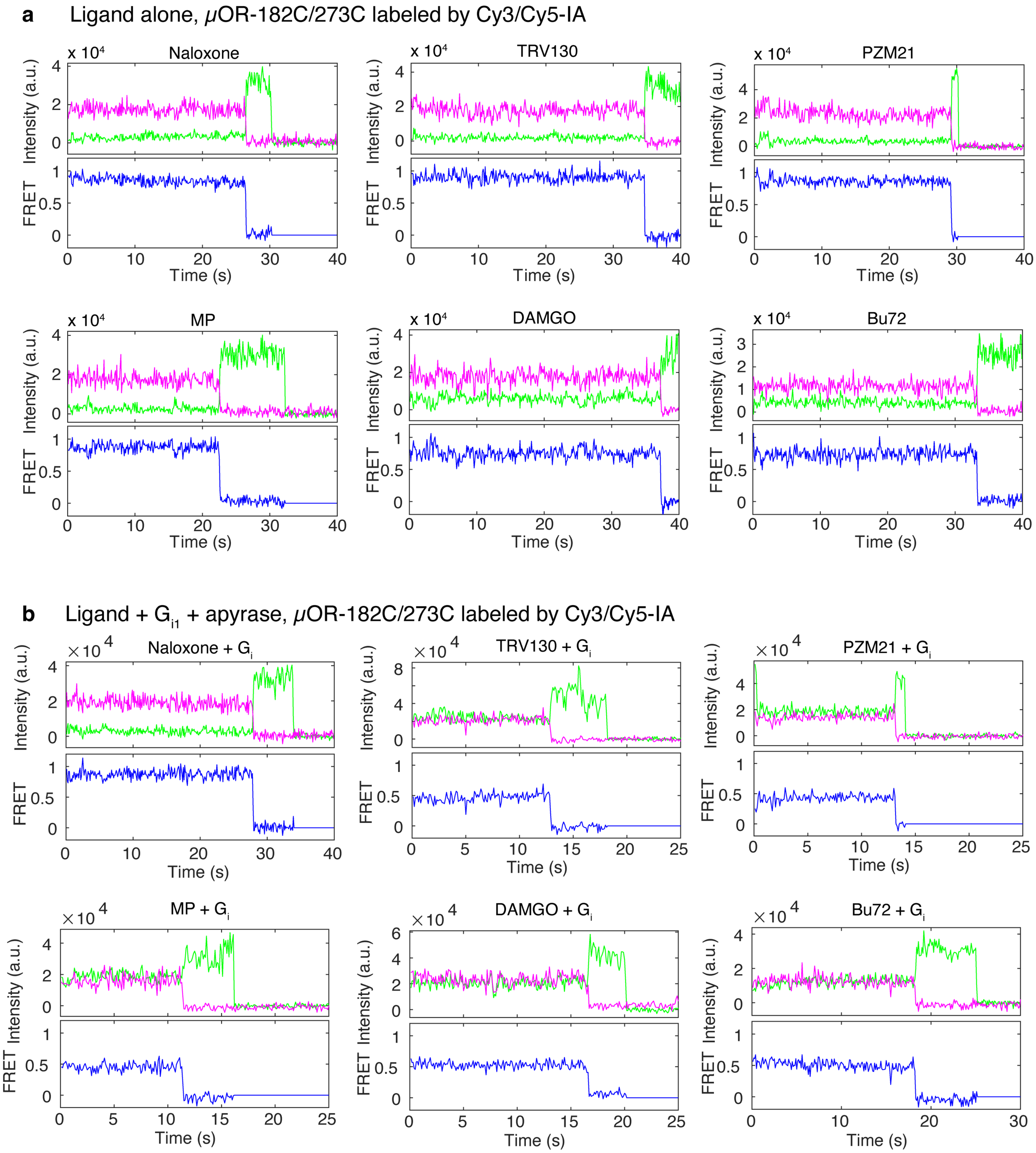

Supplementary Fig. 4. Representative fluorescence traces of Cy3 and Cy5 labeled μORΔ7-182C/273C (μOR-Cy3/Cy5). a, Fluorescence traces of μOR-Cy3/Cy5 in the presence of saturating ligands (related to Fig. 3b). **b**, Fluorescence traces of μOR-Cy3/Cy5 in the presence of saturating ligands and G_i, which were treated with apyrase to remove free GDP (related to Fig. 4a).

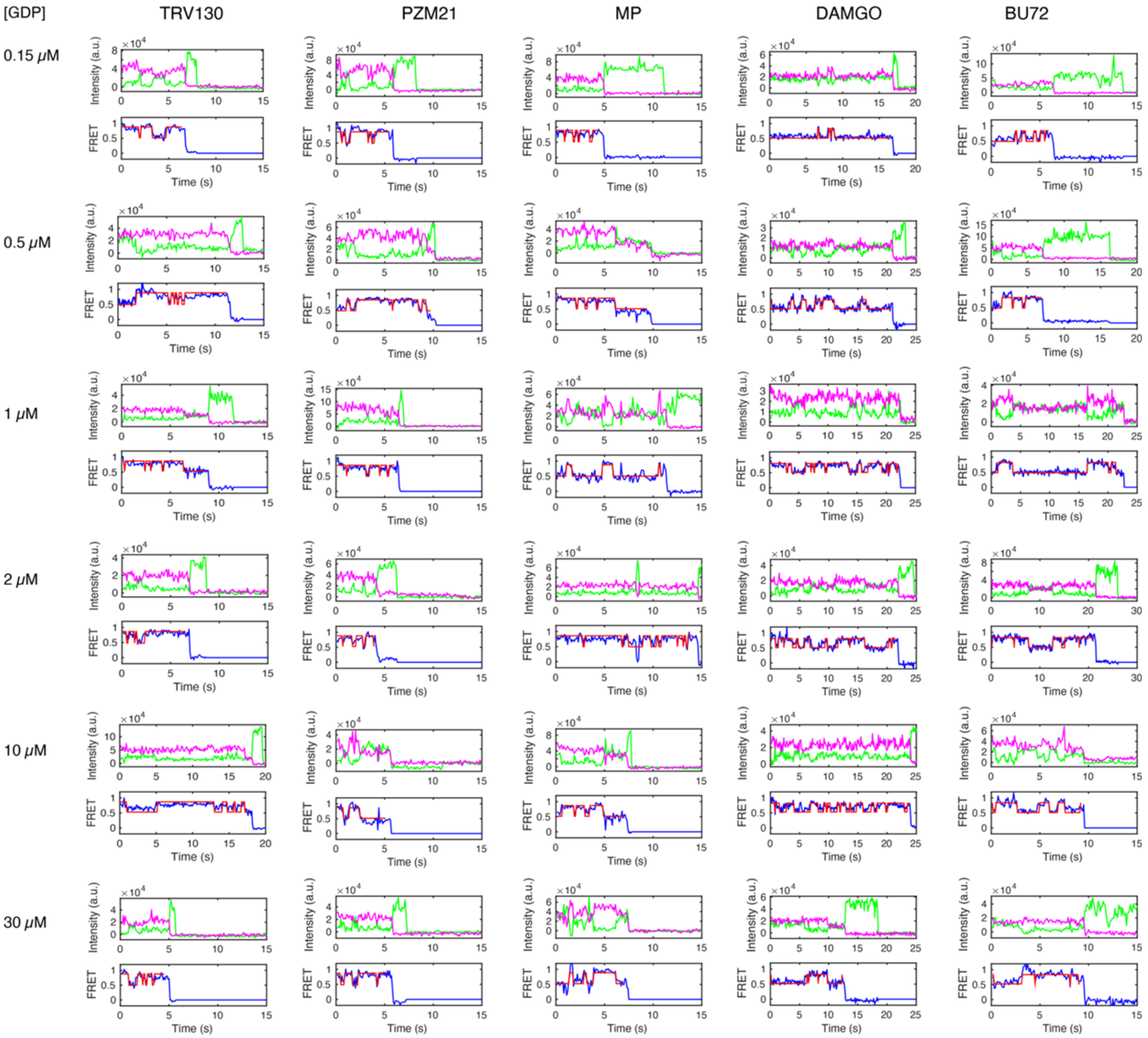

Supplementary Fig. 5. Exemplary smFRET traces and transitions of μOR-Cy3/Cy5 in the presence of 20 μM with different GDP concentrations.

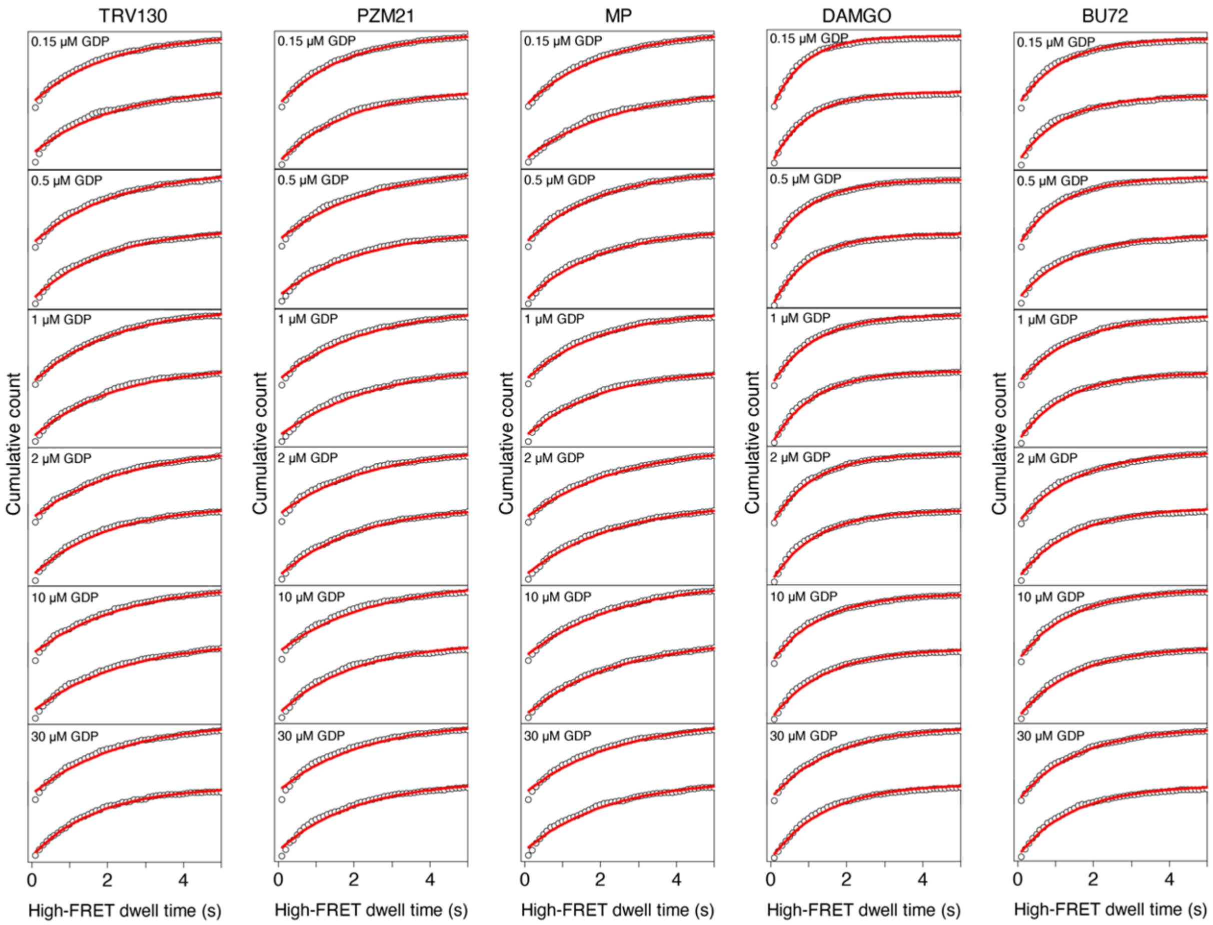

Supplementary Fig. 6. Fitting high-FRET dwell time. Cumulative counts are shown as black circles. High-FRET dwell times are fitted in single exponential decays (solid lines in red). There are two repeats for each condition.

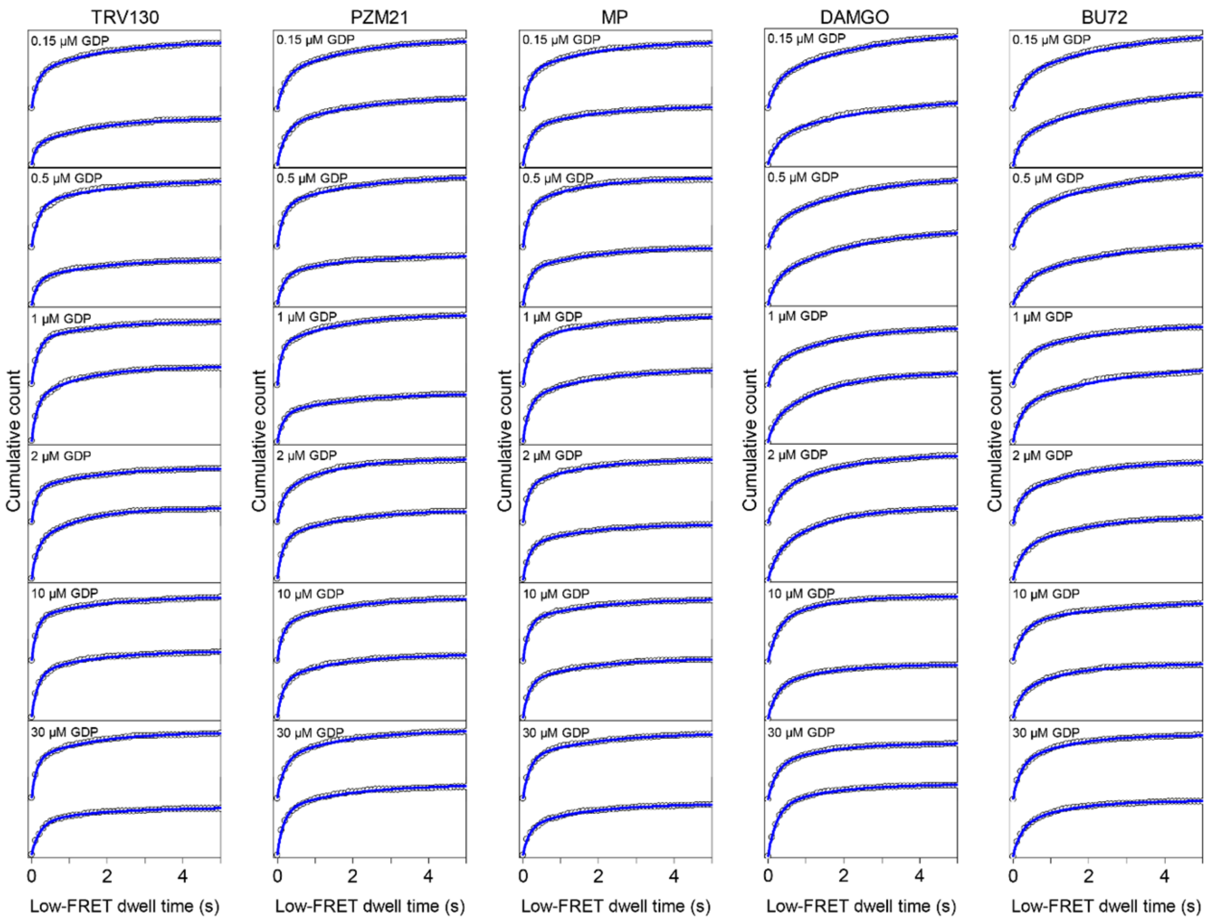

Supplementary Fig. 7. Fitting low-FRET dwell time. Cumulative counts of low-FRET dwell time for each condition are shown as black circles. Low-FRET dwell times are fitted in double exponential decays (solid lines in blue). There are two repeats for each condition.

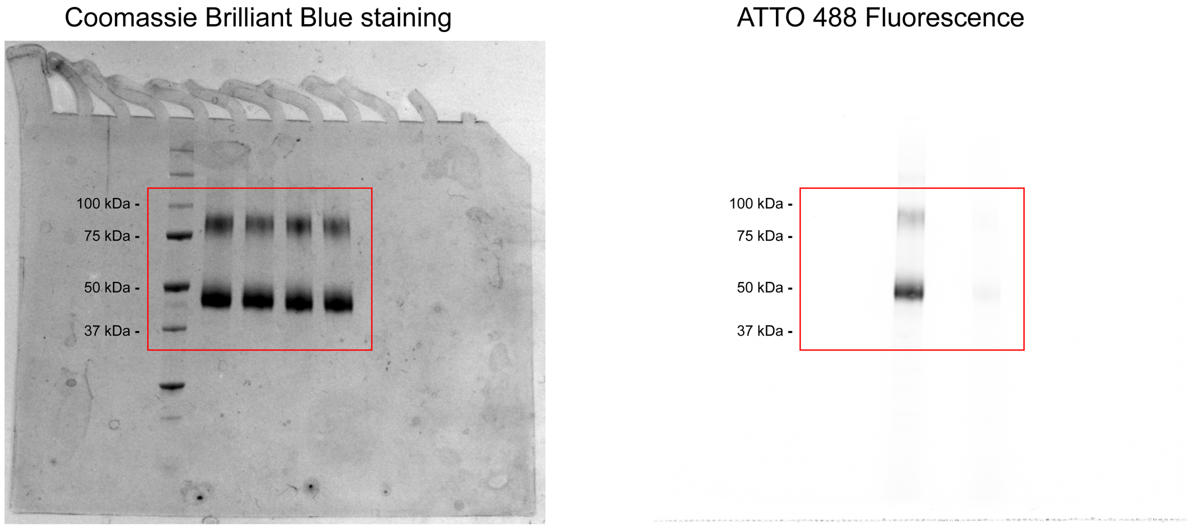

Supplementary Fig. 8. The raw, uncropped gel images for Extended Data Fig. 3a. Regions shown in Extended Data Fig. 3a are marked by red rectangles.
