# Supplementary material for: Ligand efficacy modulates conformational dynamics of the µ-opioid receptor
Source: Nature. 2024 Apr 10;629(8011):474–80. doi: 10.1038/s41586-024-07295-2 (PMC11078757; doi:10.1038/s41586-024-07295-2)
Supplement: Supplementary file 2 — Reporting Summary [file 41586_2024_7295_MOESM2_ESM.pdf]

nature portfolio

Corresponding author(s): Matthias Elgeti, Brian K. Kobilka and Chunlai Chen

Last updated by author(s): 02/15/2024

Reporting Summary

Nature Portfolio wishes to improve the reproducibility of the work that we publish. This form provides structure for consistency and transparency in reporting. For further information on Nature Portfolio policies, see our Editorial Policies and the Editorial Policy Checklist.

Statistics

For all statistical analyses, confirm that the following items are present in the figure legend, table legend, main text, or Methods section.

n/a	Confirmed	
☐	☒	The exact sample size (n) for each experimental group/condition, given as a discrete number and unit of measurement
☐	☒	A statement on whether measurements were taken from distinct samples or whether the same sample was measured repeatedly
☐	☒	The statistical test(s) used AND whether they are one- or two-sided<br>*Only common tests should be described solely by name; describe more complex techniques in the Methods section.*
☒	☐	A description of all covariates tested
☐	☒	A description of any assumptions or corrections, such as tests of normality and adjustment for multiple comparisons
☐	☒	A full description of the statistical parameters including central tendency (e.g. means) or other basic estimates (e.g. regression coefficient) AND variation (e.g. standard deviation) or associated estimates of uncertainty (e.g. confidence intervals)
☐	☒	For null hypothesis testing, the test statistic (e.g. F, t, r) with confidence intervals, effect sizes, degrees of freedom and P value noted<br>*Give P values as exact values whenever suitable.*
☒	☐	For Bayesian analysis, information on the choice of priors and Markov chain Monte Carlo settings
☒	☐	For hierarchical and complex designs, identification of the appropriate level for tests and full reporting of outcomes
☒	☐	Estimates of effect sizes (e.g. Cohen's d, Pearson's r), indicating how they were calculated

Our web collection on statistics for biologists contains articles on many of the points above.

Software and code

Policy information about availability of computer code

Data collection	Single-molecule fluorescence data was collected using Cell Vision software V1.4.0 (Beijing Coolight Technology), DEER data was collected in Xepr v2.6b.163 (Bruker, Ettlingen, Germany)
Data analysis	We used the following published software and web based analysis tools to analysis our data as referenced in methods: Origin 9.8.0.200 (OriginLab); ImageJ v1.43u; Matlab R2017a; HaMMy v4.0; DEERlab v0.9.2; LongDistances v.946;

For manuscripts utilizing custom algorithms or software that are central to the research but not yet described in published literature, software must be made available to editors and reviewers. We strongly encourage code deposition in a community repository (e.g. GitHub). See the Nature Portfolio guidelines for submitting code & software for further information.

Data

Policy information about availability of data

All manuscripts must include a data availability statement. This statement should provide the following information, where applicable:

- Accession codes, unique identifiers, or web links for publicly available datasets
- A description of any restrictions on data availability
- For clinical datasets or third party data, please ensure that the statement adheres to our policy

All the data are available in the manuscript or supplementary materials, or from the corresponding authors upon reasonable request. Raw DEER data is available at

https://zenodo.org/doi/10.5281/zenodo.10631251. Protein structure demonstration and analysis in this study used the models available at RCSB PDB (https://www.rcsb.org/) through PDB codes of 4DKL (inactive µOR) and 6DDF (active µOR).

Human research participants

Policy information about studies involving human research participants and Sex and Gender in Research.

Reporting on sex and gender	N/A
Population characteristics	N/A
Recruitment	N/A
Ethics oversight	N/A

Note that full information on the approval of the study protocol must also be provided in the manuscript.

Field-specific reporting

Please select the one below that is the best fit for your research. If you are not sure, read the appropriate sections before making your selection.

☒ Life sciences ☐ Behavioural & social sciences ☐ Ecological, evolutionary & environmental sciences

For a reference copy of the document with all sections, see nature.com/documents/nr-reporting-summary-flat.pdf

Life sciences study design

All studies must disclose on these points even when the disclosure is negative.

Sample size	Sample size for TIRF based fluorescence imaging was the number of individual molecules. Therefore, in most conditions, approximately, >300 individual molecules were analyzed, which are sufficient for statistical analysis. The fluorescence experiments were repeated 3+ times to allow calculation of the mean and standard error of the mean. For functional assays (such as BRET1 and BRET2), there are at least three biological replicates that are reported in the figure legends.<br>No sample size calculation was performed. The sample sizes are sufficient since each experiment was carried out with controls and replicated more than once.
Data exclusions	No data were excluded from the analysis.
Replication	All smFRET results were successfully reproduced through at least 2-4 independent attempts.<br>DEER data was collected for several different constructs and different spin labels using a limited set of conditions (Apo, naloxone, MP, morphine, PZM21, DAMGO, lofentanil, DAMGO + Gi). After optimizing construct and label, we acquired the full set of conditions reported here. For functional assay, data were replicated using three biological replicates. See figure legends for specific details.
Randomization	Not relevant to this study.
Blinding	Blinding was not possible, however, appropriate controls were used during experiments.

Reporting for specific materials, systems and methods

We require information from authors about some types of materials, experimental systems and methods used in many studies. Here, indicate whether each material, system or method listed is relevant to your study. If you are not sure if a list item applies to your research, read the appropriate section before selecting a response.

Materials & experimental systems

n/a	Involved in the study	
☐	☒	Antibodies
☐	☒	Eukaryotic cell lines
☒	☐	Palaeontology and archaeology
☒	☐	Animals and other organisms
☒	☐	Clinical data
☒	☐	Dual use research of concern

Methods

n/a	Involved in the study	
☒	☐	ChIP-seq
☒	☐	Flow cytometry
☒	☐	MRI-based neuroimaging

Antibodies

Antibodies used	Monoclonal (clone M1) ANTI-FLAG antibody produced in mouse (Sigma, Cat. No. F3040). The antibody was not directly used, but was processed to generate Fab, which was then biotinylated.
Validation	Antibody was validated by the commercial supplier. The statement can be found on the website: https://www.sigmaaldrich.com/US/en/product/sigma/f3040

Eukaryotic cell lines

Policy information about cell lines and Sex and Gender in Research

Cell line source(s)	Sf9 and Hi5 cell lines were from Expression Systems, LLC (USA). Source for HEK 293T cell line is ATCC.
Authentication	The cell lines were not authenticated.
Mycoplasma contamination	The cell lines were not tested for mycoplasma contamination.
Commonly misidentified lines (See ICLAC register)	No commonly misidentified cell lines were used.
